# Supplementary material for: Diagnostic Value of Menstrual Blood Lipidomics in Endometriosis: A Pilot Study
Source: Biomolecules. 2024 Jul 24;14(8):899. doi: 10.3390/biom14080899 (PMC11351896; doi:10.3390/biom14080899)
Supplement: Supplementary file 1 [file biomolecules-14-00899-s001.zip › supplementary2_biomolecules.pdf]

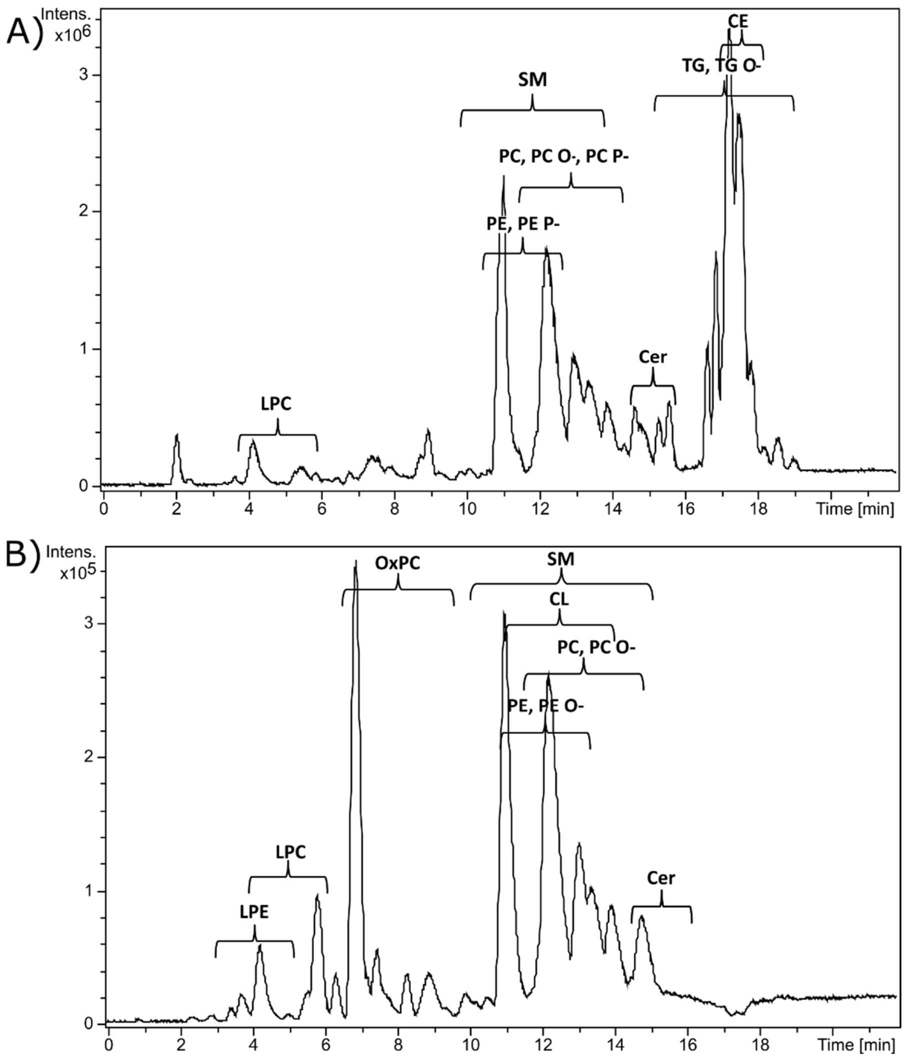

Figure S1. Based ion chromatogram of dry blood spot lipids profile in A) positive ion mode, B) negative ion mode. CE – cholesterol ester, Cer – ceramide, CL – cardiolipine, LPC – lysophosphatidylcholine, LPE – lysophosphatidylethanolamine, PC – phosphatidylcholine, PC O- -plasmanylphosphatidylcholines, PC P- - plasmenylphosphatidylcholines, PE – phoshpatidylethanolamine, PE O- - plasmanylphosphatidylethanolamine, PE P- - plasmenylethanolamine, SM – sphingomyeline, TG – triacylglycerol, TG O- - plasmanyltriacylglycerol.

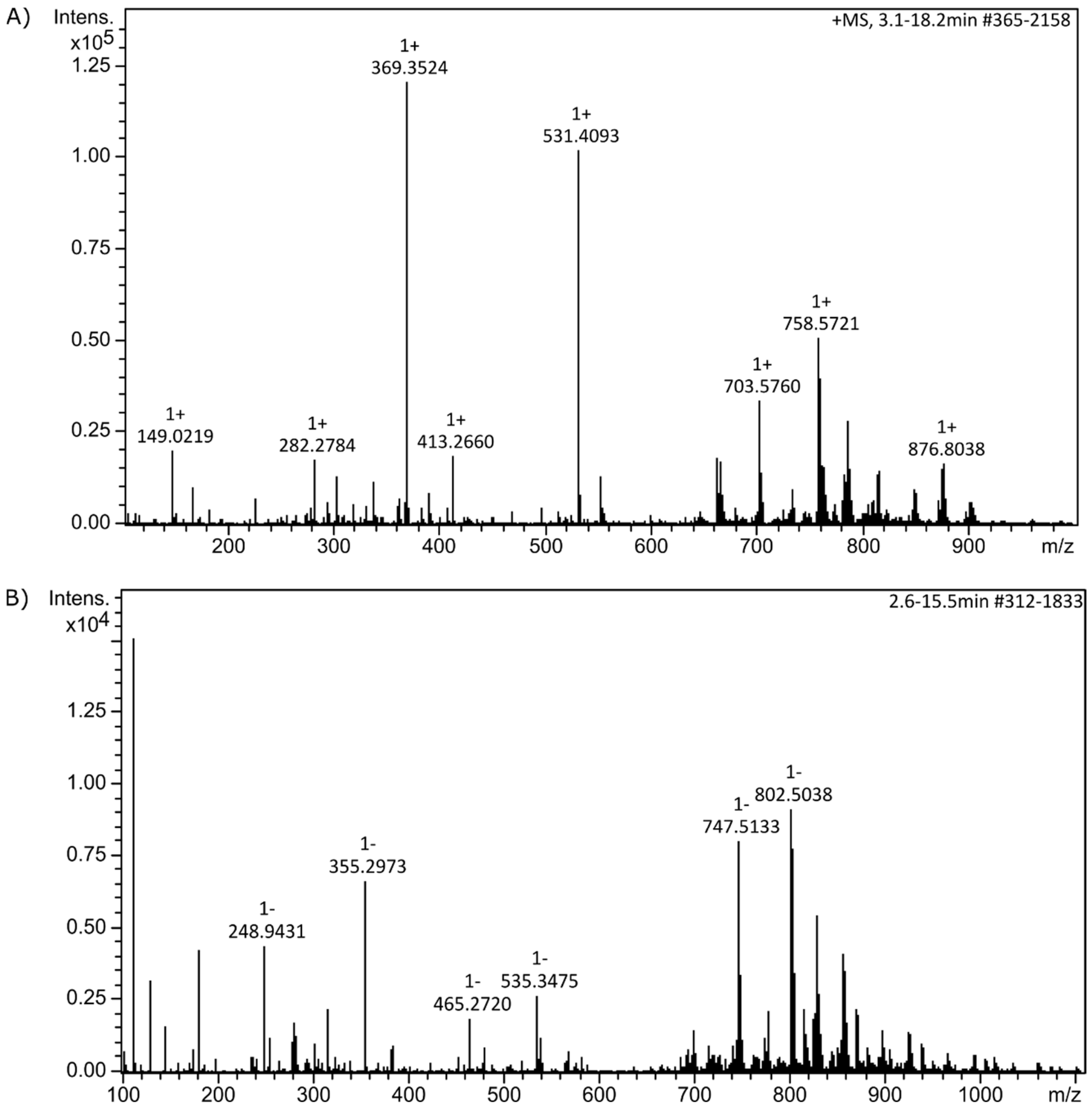

Figure S2. Average mass spectra of dried menstrual blood spot lipid profile. A) Positive ion mode. B) Negative ion mode.

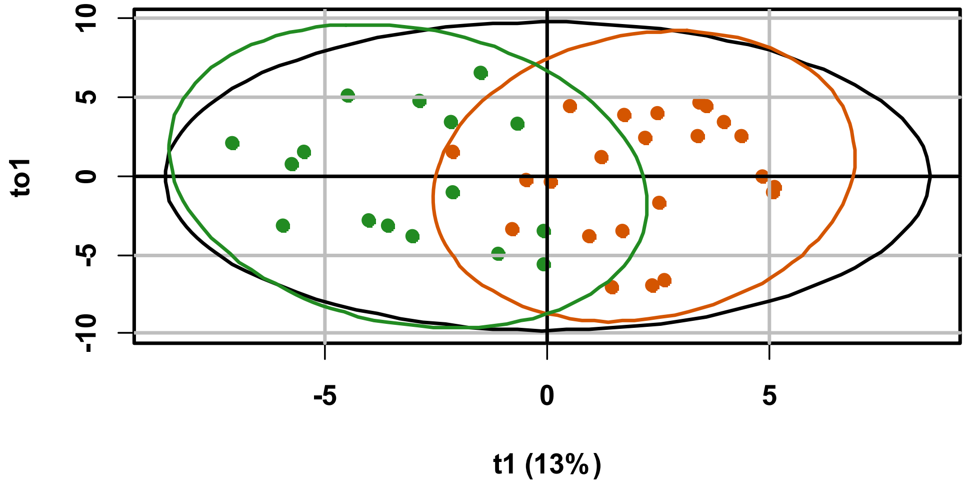

Figure S3. Score plot of samples in space "predictive component of OPLS model – orthogonal component of OPLS model".

Table S1. Statistically significant (FDR<0.05) enriched pathways in endometriosis.

Pathway	Database	Size of pathway (metabolites)	Classes of markers, included in pathway	P	FDR
**MTHFR deficiency**	Wikipathways	21	Cer-NS; SM; PC; PE-P	<0.001	<0.001
**Glycerophospholipid biosynthesis**	Reactome	94	PC-P; TG; CL; PC; PE-P	<0.001	<0.001
**Phospholipid metabolism**	Reactome	104	PC-P; TG; CL; PC; PE-P	<0.001	<0.001
**Metabolism of lipids**	Reactome	626	TG; CL; PC; PE-P; Cer-NS; PC-P; SM	<0.001	<0.001
**Sphingolipid de novo biosynthesis**	Reactome	35	Cer-NS; SM; PC; PE-P	<0.001	<0.001
**Glycerophospholipid metabolism**	EHMN	95	PC-P; TG; CL; PC; PE-P	<0.001	<0.001
**Oligodendrocyte specification and differentiation, leading to myelin components for CNS**	Wikipathways	11	SM; PC; PE-P	<0.001	<0.001
**Acyl chain remodeling of CL**	Reactome	12	CL; PC; PE-P	<0.001	<0.001
**Sphingolipid metabolism**	Reactome	65	Cer-NS; SM; PC; PE-P	<0.001	<0.001
**ceramide signaling pathway**	BioCarta	2	Cer-NS; SM	<0.001	<0.001
**Immune System**	Reactome	147	Cer-NS; TG; PC; PE-P	<0.001	<0.001
**Ceramide signalling**	Reactome	5	Cer-NS; SM	<0.001	<0.001
**Synthesis of PS**	Reactome	6	PC; PE-P	<0.001	<0.001
**Metabolism**	Reactome	1411	TG; CL; PC; PE-P; Cer-NS; PC-P; SM	<0.001	<0.001
**Sphingolipid Metabolism**	SMPDB	40	Cer-NS; SM; PC	<0.001	<0.001
**Gaucher Disease**	SMPDB	40	Cer-NS; SM; PC	<0.001	<0.001
**Globoid Cell Leukodystrophy**	SMPDB	40	Cer-NS; SM; PC	<0.001	<0.001
**Metachromatic Leukodystrophy (MLD)**	SMPDB	40	Cer-NS; SM; PC	<0.001	<0.001
**Fabry disease**	SMPDB	40	Cer-NS; SM; PC	<0.001	<0.001
**Krabbe disease**	SMPDB	40	Cer-NS; SM; PC	<0.001	<0.001
**Neurodegeneration with brain iron accumulation (NBIA) subtypes pathway**	Wikipathways	23	Cer-NS; SM	<0.001	<0.001
**Immunoregulatory interactions between a Lymphoid and a non-Lymphoid cell**	Reactome	20	Cer-NS; PE-P	<0.001	<0.001
**Formation of the cornified envelope**	Reactome	10	Cer-NS; TG	<0.001	<0.001
**Keratinization**	Reactome	10	Cer-NS; TG	<0.001	<0.001
**Glycerophospholipid metabolism - Homo sapiens (human)**	KEGG	55	CL; PC; PE-P	<0.001	<0.001
**Acyl chain remodelling of PE**	Reactome	13	PC; PE-P	<0.001	<0.001
**Necroptosis - Homo sapiens (human)**	KEGG	10	Cer-NS; SM	<0.001	<0.001

**ABC transporters in lipid homeostasis**	Reactome	11	SM; PC	<0.001	<0.001
**p75 NTR receptor-mediated signalling**	Reactome	11	Cer-NS; SM	<0.001	<0.001
**Acyl chain remodelling of PC**	Reactome	13	PC-P; PC	<0.001	<0.001
**Death Receptor Signalling**	Reactome	12	Cer-NS; SM	<0.001	<0.001
**Transport of small molecules**	Reactome	219	TG; SM; PC; PE-P	<0.001	0.001
**HDL remodeling**	Reactome	13	TG; PC	<0.001	0.001
**Plasma lipoprotein remodeling**	Reactome	16	TG; PC	<0.001	0.002
**Sphingolipid signaling pathway - Homo sapiens (human)**	KEGG	15	Cer-NS; SM	<0.001	0.002
**ABC-family proteins mediated transport**	Reactome	20	SM; PC	<0.001	0.002
**Plasma lipoprotein assembly, remodeling, and clearance**	Reactome	21	TG; PC	<0.001	0.003
**Retrograde endocannabinoid signaling - Homo sapiens (human)**	KEGG	19	PC; PE-P	<0.001	0.003
**Insulin resistance - Homo sapiens (human)**	KEGG	20	Cer-NS; TG	<0.001	0.003
**Phospholipid Biosynthesis**	SMPDB	27	PC; PE-P	<0.001	0.003
**Synthesis of PC**	Reactome	28	PC; PE-P	<0.001	0.003
**Developmental Biology**	Reactome	30	Cer-NS; TG	<0.001	0.004
**Synthesis of PA**	Reactome	27	CL; PC	<0.001	0.004
**Sphingolipid metabolism - Homo sapiens (human)**	KEGG	25	Cer-NS; SM	<0.001	0.004
**Membrane Trafficking**	Reactome	31	TG; PC	<0.001	0.005
**sphingomyelin metabolism/ceramide salvage**	HumanCyc	55	Cer-NS; PC	0.001	0.007
**Adaptive Immune System**	Reactome	51	Cer-NS; PE-P	0.001	0.007
**Glycosphingolipid metabolism**	Reactome	38	Cer-NS; SM	0.001	0.007
**TNF receptor signaling pathway**	PID	1	Cer-NS	0.002	0.008
**fas signaling pathway (cd95)**	BioCarta	1	Cer-NS	0.002	0.008
**Pathogenic Escherichia coli infection**	Wikipathways	1	PE-P	0.002	0.008
**Host-pathogen interaction of human coronaviruses - autophagy**	Wikipathways	1	PE-P	0.002	0.008
**FOXA2 pathway**	Wikipathways	1	TG	0.002	0.008
**Glycerophospholipid Biosynthetic Pathway**	Wikipathways	49	PC-P; CL	0.002	0.008
**One-carbon metabolism and related pathways**	Wikipathways	42	PC; PE-P	0.002	0.009
**Vesicle-mediated transport**	Reactome	66	TG; PC	0.003	0.01
**Visual phototransduction**	Reactome	68	TG; PE-P	0.003	0.01
**Sensory Perception**	Reactome	69	TG; PE-P	0.003	0.01
**Glycosphingolipid metabolism**	EHMN	52	Cer-NS; SM	0.003	0.01
**multi-drug resistance factors**	BioCarta	2	PC	0.003	0.01
**ras signaling pathway**	BioCarta	2	PC	0.003	0.01
**PI and PC transport between ER and Golgi membranes**	Reactome	2	PC	0.003	0.01

**TRAIL signaling pathway**	PID	2	Cer-NS	0.003	0.01
**inactivation of gsk3 by akt causes accumulation of b-catenin in alveolar macrophages**	BioCarta	2	Cer-NS	0.003	0.01
**FAS (CD95) signaling pathway**	PID	2	Cer-NS	0.003	0.01
**TNF-alpha signaling pathway**	Wikipathways	3	Cer-NS	0.003	0.01
**Autophagy**	Wikipathways	2	PE-P	0.003	0.01
**phosphatidylserine biosynthesis I**	HumanCyc	4	PC	0.005	0.02
**p75(NTR)-mediated signaling**	PID	3	Cer-NS	0.005	0.02
**Ceramide signaling pathway**	PID	3	Cer-NS	0.005	0.02
**Pathogenic Escherichia coli infection - Homo sapiens (human)**	KEGG	3	PE-P	0.005	0.02
**Autophagy - other - Homo sapiens (human)**	KEGG	3	PE-P	0.005	0.02
**Lipid particle organization**	Reactome	3	TG	0.005	0.02
**glutathione redox reactions I**	HumanCyc	100	Cer-NS; PC	0.005	0.02
**Signal Transduction**	Reactome	290	Cer-NS; SM; PC	0.005	0.02
**Innate Immune System**	Reactome	119	TG; PC	0.005	0.02
**regulation of bad phosphorylation**	BioCarta	4	Cer-NS	0.006	0.02
**Biosynthesis of A2E, implicated in retinal degradation**	Reactome	7	PE-P	0.006	0.02
**Retinoid cycle disease events**	Reactome	7	PE-P	0.006	0.02
**Diseases associated with visual transduction**	Reactome	7	PE-P	0.006	0.02
**Diseases of the neuronal system**	Reactome	7	PE-P	0.006	0.02
**Chylomicron assembly**	Reactome	4	TG	0.006	0.02
**VLDL assembly**	Reactome	4	TG	0.006	0.02
**LDL remodeling**	Reactome	4	TG	0.006	0.02
**Chylomicron clearance**	Reactome	4	TG	0.006	0.02
**VLDL clearance**	Reactome	4	TG	0.006	0.02
**HDL clearance**	Reactome	4	TG	0.006	0.02
**Assembly of active LPL and LIPC lipase complexes**	Reactome	6	TG	0.006	0.02
**Synthesis of CL**	Reactome	4	CL	0.006	0.02
**Neurotrophin signaling pathway - Homo sapiens (human)**	KEGG	5	Cer-NS	0.008	0.02
**Nanoparticle triggered regulated necrosis**	Wikipathways	8	Cer-NS	0.008	0.02
**Kaposi sarcoma-associated herpesvirus infection - Homo sapiens (human)**	KEGG	5	PE-P	0.008	0.02
**Scavenging by Class F Receptors**	Reactome	9	TG	0.008	0.02
**Composition of Lipid Particles**	Wikipathways	10	TG	0.008	0.02
**Pyroptosis**	Reactome	5	CL	0.008	0.02

**Leishmaniasis - Homo sapiens (human)**	KEGG	6	Cer-NS	0.01	0.02
**IL2 signaling events mediated by PI3K**	PID	7	Cer-NS	0.01	0.02
**metabolism of anandamide an endogenous cannabinoid**	BioCarta	6	PE-P	0.01	0.02
**Autophagy - animal - Homo sapiens (human)**	KEGG	6	PE-P	0.01	0.02
**Platelet sensitization by LDL**	Reactome	6	TG	0.01	0.02
**Chylomicron remodeling**	Reactome	7	TG	0.01	0.02
**Regulation of TLR by endogenous ligand**	Reactome	13	TG	0.01	0.02
**Phosphatidylcholine catabolism**	Wikipath ways	9	SM	0.01	0.02
**phospho-PLA2 pathway**	Reactome	7	PC	0.01	0.03
**Adipocytokine signaling pathway - Homo sapiens (human)**	KEGG	7	Cer-NS	0.01	0.03
**Degradation pathway of sphingolipids, including diseases**	Wikipath ways	13	Cer-NS	0.01	0.03
**Triacylglyceride synthesis**	Wikipath ways	10	TG	0.01	0.03
**phospholipids as signalling intermediaries**	BioCarta	7	SM	0.01	0.03
**phosphatidylcholine biosynthesis pathway**	BioCarta	8	PC	0.01	0.03
**Fc-epsilon receptor I signaling in mast cells**	PID	8	PC	0.01	0.03
**COPI-independent Golgi-to-ER retrograde traffic**	Reactome	9	PC	0.01	0.03
**Heme signaling**	Reactome	10	TG	0.01	0.03
**Scavenging by Class H Receptors**	Reactome	11	TG	0.01	0.03
**Acetylcholine Synthesis**	Wikipath ways	11	PC	0.01	0.03
**AGE-RAGE signaling pathway in diabetic complications - Homo sapiens (human)**	KEGG	9	Cer-NS	0.01	0.03
**LDL clearance**	Reactome	9	TG	0.01	0.03
**Plasma lipoprotein clearance**	Reactome	9	TG	0.01	0.03
**Scavenging by Class B Receptors**	Reactome	16	TG	0.01	0.03
**Golgi-to-ER retrograde transport**	Reactome	11	PC	0.02	0.03
**Macroautophagy**	Reactome	12	PE-P	0.02	0.03
**Cargo recognition for clathrin-mediated endocytosis**	Reactome	10	TG	0.02	0.03
**HIF1A and PPARG regulation of glycolysis**	Wikipath ways	10	TG	0.02	0.03
**Acyl chain remodeling of DAG and TAG**	Reactome	10	TG	0.02	0.03
**Cholesterol metabolism - Homo sapiens (human)**	KEGG	10	TG	0.02	0.03
**Ca-dependent events**	Reactome	11	PC	0.02	0.03
**Choline metabolism in cancer - Homo sapiens (human)**	KEGG	11	PC	0.02	0.03

**phosphatidylcholine biosynthesis**	HumanCyc	12	PC	0.02	0.03
**Autophagy**	Reactome	13	PE-P	0.02	0.03
**Plasma lipoprotein assembly**	Reactome	11	TG	0.02	0.03
**Cell surface interactions at the vascular wall**	Reactome	13	TG	0.02	0.03
**Statin inhibition of cholesterol production**	Wikipathways	17	TG	0.02	0.03
**Intra-Golgi and retrograde Golgi-to-ER traffic**	Reactome	13	PC	0.02	0.04
**Visual signal transduction: Rods**	PID	13	PC	0.02	0.04
**Role of phospholipids in phagocytosis**	Reactome	13	PC	0.02	0.04
**Alpha Linolenic Acid and Linoleic Acid Metabolism**	SMPDB	18	PC	0.02	0.04
**Sphingolipid pathway**	Wikipathways	14	Cer-NS	0.02	0.04
**Lipid Metabolism Pathway**	Wikipathways	14	TG	0.02	0.04
**Toll-like Receptor Cascades**	Reactome	31	TG	0.02	0.04
**Surfactant metabolism**	Reactome	13	PC	0.02	0.04
**Synthesis of PG**	Reactome	13	PC	0.02	0.04
**Visual signal transduction: Cones**	PID	14	PC	0.02	0.04
**Serine Metabolism**	Wikipathways	27	Cer-NS	0.02	0.04
**Fat digestion and absorption - Homo sapiens (human)**	KEGG	13	TG	0.02	0.04
**16p11.2 proximal deletion syndrome**	Wikipathways	40	Cer-NS	0.02	0.04
**Glycosylphosphatidylinositol(GPI)-anchor biosynthesis**	EHMN	14	PE-P	0.02	0.04
**Ion transport by P-type ATPases**	Reactome	17	PE-P	0.02	0.04
**Lipid and atherosclerosis - Homo sapiens (human)**	KEGG	14	TG	0.02	0.04
**Regulation of lipolysis in adipocytes - Homo sapiens (human)**	KEGG	14	TG	0.02	0.04
**Regulated Necrosis**	Reactome	16	CL	0.02	0.04
**Fcgamma receptor (FCGR) dependent phagocytosis**	Reactome	16	PC	0.02	0.04
**ceramide *de novo* biosynthesis**	HumanCyc	20	Cer-NS	0.03	0.04
**Glycosylphosphatidylinositol (GPI)-anchor biosynthesis - Homo sapiens (human)**	KEGG	16	PE-P	0.03	0.04
**Kennedy pathway from sphingolipids**	Wikipathways	18	PE-P	0.03	0.04
**Synthesis of glycosylphosphatidylinositol (GPI)**	Reactome	19	PE-P	0.03	0.04
**Clathrin-mediated endocytosis**	Reactome	17	TG	0.03	0.04
**Triglyceride biosynthesis**	Reactome	18	TG	0.03	0.04

**PLC beta mediated events**	Reactome	17	PC	0.03	0.04
**G-protein mediated events**	Reactome	17	PC	0.03	0.04
**Digestion of dietary lipid**	Reactome	17	TG	0.03	0.04
**Triglyceride catabolism**	Reactome	19	TG	0.03	0.04
**Ion channel transport**	Reactome	22	PE-P	0.03	0.04
**Thyroid hormones production and their peripheral downstream signaling effects**	Wikipath ways	25	TG	0.03	0.04
**Programmed Cell Death**	Reactome	21	CL	0.03	0.04
**Post-translational modification: synthesis of GPI-anchored proteins**	Reactome	22	PE-P	0.03	0.047
**Metabolism of Spingolipids in ER and Golgi apparatus**	Wikipath ways	21	Cer-NS	0.03	0.049
**Synthesis of PE**	Reactome	21	PE-P	0.03	0.049
**Scavenging by Class A Receptors**	Reactome	32	TG	0.03	0.049
